# Exchange rates of second generation Microcuff® pediatric endotracheal tubes in children weighing more than 3 kg: A retrospective audit

**DOI:** 10.1007/s00101-024-01486-2

**Published:** 2024-11-28

**Authors:** Ilka Schmidt-Deubig, Michael Kemper, Pedro D. Wendel-Garcia, Markus Weiss, Jörg Thomas, Christian Peter Both, Achim Schmitz

**Affiliations:** 1 Department of Anesthesia, University Children’s Hospital, Lenggstrasse 30, 8008 Zurich, Switzerland; 2 Department of Anesthesiology, RWTH University Hospital, Aachen, Germany; 3 Institute of Intensive Care Medicine, University Hospital, Zurich, Switzerland

**Keywords:** Pediatric anesthesia, Cuffed endotracheal tubes, Tube exchange, Reintubation, Tube size selection, Kinderanästhesie, Gecuffte Endotrachealtuben, Tubuswechsel, Reintubation, Tubusgrößenauswahl

## Abstract

**Background:**

Cuffed endotracheal tubes (cETT) pose the potential advantage of an infrequent need for reintubation in pediatric patients compared to uncuffed tubes. The aim of this study was to investigate tube exchange rates using second generation Microcuff® pediatric endotracheal tubes (PET) with an adapted sizing recommendation in a large single institution cohort of children and to identify potential variables associated with an elevated risk of tube exchange.

**Methods:**

Patient data obtained from the electronic patient data management system of the Department of Anesthesia, University Children’s Hospital Zurich, Switzerland, were retrospectively assessed for demographic and anthropometric information, size of the internal tube diameter used for positive pressure ventilation and divergence from the size recommendation chart.

**Results:**

Data from 14,188 children younger than 16 years (median 5.3 years) and weighing at least 3 kg who underwent oral or nasal tracheal intubation using second generation Microcuff® PET between 2009 and 2015 were included. Of 13,219 oral tracheal intubations 12,049 (84.9%) were performed according to the manufacturer’s size recommendation and 1170 with divergent endotracheal tubes. The odds ratio (OR) of oral reintubation was 0.13% (95% confidence interval 0.08–0.22%) for cases using the manufacture’s size recommendation correctly and 22.74% (95% confidence interval 20.42–25.23%) for patients intubated with a not recommended tube (*p* < 0.0001).

**Conclusion:**

These findings indicate that the second generation Microcuff® PETs can be reliably used with low tube exchange rates across the entire pediatric age range when the tube size is selected according to the manufacturer’s size recommendation chart. Adherence to the manufacturer’s tube size recommendation is urgently advised.

## Abstract (de)

**Hintergrund:**

Gecuffte Endotrachealtuben (gETT) bieten bei pädiatrischen Patienten im Vergleich zu nichtgecufften Endotrachealtuben (nETT) mehrere wichtige Vorteile. So ist das Risiko von Mikroaspirationen bei der Langzeitbeatmung von Kindern auf der Intensivstation deutlich geringer und das endtidale CO_2_ kann präziser bestimmt und damit die Beatmung besser gesteuert werden. Zudem ist die Rate notwendiger Endotrachealtubus(ETT)-Wechsel beispielsweise aufgrund von zu großen Undichtigkeiten deutlich geringer. Dies stellt einen wesentlichen Vorteil des gETT gegenüber dem nETT im operativen Bereich dar. Dieser wichtige Vorteil wurde in mehreren Studien für gecuffte pädiatrische Microcuff®-Endotrachealtuben (Microcuff®-PET) der ersten Generation gezeigt. Ziel dieser Studie war es nun, die Tubuswechselraten bei Verwendung von Microcuff®-PET der zweiten Generation mit einer angepassten Größenempfehlung in einer großen Kohorte von Kindern an einer großen Kinderklinik zu untersuchen und Variablen zu identifizieren, die mit dem erhöhten Risiko eines Tubuswechsels verbunden sein könnten.

**Methoden:**

Aus dem elektronischen Patientendatenmanagementsystem der Abteilung für Anästhesie des Universitäts-Kinderspitals Zürich, Schweiz, wurden Patientendaten retrospektiv in Bezug auf demografische und anthropometrische Informationen, die Größe des verwendeten Tubusinnendurchmessers für die invasive Beatmung, sowie Abweichungen von der empfohlenen Tubusgrößenliste ausgewertet.

**Ergebnisse:**

Daten von 14.188 Kindern unter 16 Jahren (Median 5,3 Jahre) mit einem Gewicht von mindestens 3 kg, die zwischen 2009 und 2015 oral oder nasal mit Microcuff®-PET der zweiten Generation intubiert wurden, wurden einbezogen. Von 13.219 orotrachealen Intubationen wurden 12.049 (84,9 %) gemäß der Größenempfehlung des Herstellers durchgeführt und 1170 mit Endotrachealtuben, die von der Empfehlung abwichen. Die Odds Ratio für eine orotracheale Reintubation betrug 0,13 % (95 % Konfidenzintervall 0,08–0,22 %) für Fälle, in denen die Größenempfehlung des Herstellers korrekt berücksichtigt wurde, und 22,74 % (95 % Konfidenzintervall 20,42–25,23 %) für Patienten, die mit einem nichtempfohlenen Tubus intubiert wurden (*p* < 0,0001).

**Schlussfolgerung:**

Diese Ergebnisse zeigen, dass Microcuff®-PET der zweiten Generation über die gesamte pädiatrische Altersspanne hinweg zuverlässig mit niedrigen Tubuswechselraten verwendet werden können, wenn die Tubusgröße entsprechend der Größenauswahltabelle des Herstellers ausgewählt wird. Die strikte Einhaltung der Größenempfehlung des Herstellers wird dringend angeraten.

## Treten Sie in den Austausch

Diese Arbeit wurde für *Die Anaesthesiologie* in Englisch eingereicht und angenommen. Die deutsche Zusammenfassung wurde daher etwas ausführlicher gestaltet. Wenn Sie über diese Zusammenfassung hinaus Fragen haben und mehr wissen wollen, nehmen Sie gern in Deutsch über die Korrespondenzadresse am Ende des Beitrags Kontakt auf. Die Autor:innen freuen sich auf den Austausch mit Ihnen.

## What is already known.

For the first generation Microcuff® pediatric endotracheal tubes (PET) introduced in 2004, very low tube exchange rates (0.0–2.6%) have been reported for healthy children of all ages weighing at least 3 kg.

## What this article adds.

Since 2007 the second generation Microcuff® PET with larger outer diameters and an adapted age-related size selection recommendation are delivered by the manufacturer. The use of second-generation Microcuff® PETs demonstrate very low tube exchange rates across the entire pediatric age range.

## Introduction

Cuffed endotracheal tubes (cETT) have significant advantages over endotracheal tubes (ETTs) without cuffs. Advantages are for example in situations of reduced pulmonary compliance, a better protection against microaspiration [1], a reduced gas leakage, a more accurate capnography and spirometry and an increased reliability in the delivery of tidal volumes [2–11]. A major advantage of cETTs in children is the considerably reduced ETT exchange rate compared to uncuffed ETT [4, 7]. Comprehensive prospective clinical and endoscopic studies have demonstrated that adequately designed cETTs used according to the manufacturer’s recommendations can be used in infants weighing ≥ 3 kg without increasing the airway morbidity [2, 4, 7, 10–13]. Thus, they are increasingly used in children, including infants and neonates, and have become the standard type of ETT in pediatric anesthesia [14].

The first generation of Microcuff® PETs, introduced in 2004, have proved to be well-fitting and sealing, with exchange rates ranging from 0.0% to 2.6% for recommended tube sizes [4, 12, 15, 16]. In two of these studies, the internal diameter (ID) 3.5 mm and 4.0 mm Microcuff® PET were even used in smaller children than currently recommended [4, 15]. The studies performed for elective procedures with the first generation Microcuff® PET mainly included healthy and fasting children.

Since 2007, second generation Microcuff® PETs with larger outer diameters reducing the risk for ETT kinking and accordingly with an adapted age-related size selection recommendation, particularly in the smaller tube sizes, are delivered by the manufacturer [17]. So far, there are no studies investigating ETT exchange rates using this altered equipment and adapted size recommendation. Therefore, this retrospective study aimed to investigate ETT exchange rates using second generation Microcuff® PETs with an adapted size recommendation in a large single institution cohort of children and to elucidate potential variables associated with an elevated risk for ETT exchange rates.

## Material and methods

This retrospective observational single center trial was approved by the regional ethics committee of Zurich, Switzerland (BASEC-No 2016-00188 with first and second amendments). The need for written informed consent by the guardian was waived by the ethics committee.

Screening of the electronic patient data management system (PDMS) of the Department of Anesthesia, University Children’s Hospital Zurich, Switzerland, was performed retrospectively for patients weighing ≥ 3 kg and requiring endotracheal intubation between January 2009 and March 2015. The PDMS software components were deioRecorder™, deioWarehouse™ and deioAnalyzer™ (Datex-Ohmeda, GE-Healthcare, Helsinki, Finland).

We included children with primary tracheal intubation in the pediatric operating theatre, cardiac catheterization laboratory, magnetic resonance imaging center or computed tomography suite, using a second generation Microcuff® PET documented as the final cETT. Both elective and emergency anesthesia cases were included. In children who underwent multiple procedures with tracheal intubation, only the first procedure was considered for statistical reasons, i.e., repetitive anesthetics were excluded.

The primary outcome parameter was the exchange rate of second generation Microcuff® PET due to the need for an alternative cETT size (nonfitting). As secondary outcome parameter, the frequency of deviation from the recommended cETT size without requiring ETT exchange was investigated.

According to the institutional guidelines, cETTs were used for tracheal intubation in children weighing 3 kg and more. For size selection of cETT the manufacturer’s size recommendation chart (SRC) was used (Table 1). Demographic and clinical data are presented as median (interquartile range, IQR) or *n* (%) in Table 2. Tracheal intubation was performed after intravenous or inhalational induction of anesthesia, following routine muscle paralysis using atracurium or mivacurium, according to the institutional standard. All tracheal intubations were performed or supervised by a consultant pediatric anesthesiologist.

**Table 1:** Manufacturer’s cETT size recommendation chart (SRC), the number of tracheal intubations, and endotracheal tube (ETT) exchange rates

Children’s age	Recommended tube size SRC ID (mm)	Number of tracheal intubations *n* (%)	Number of final tubes appropriate to SRC *n* (%)	ETT exchange negative *n* (%)
≥ 3 kg body weight to < 8 months	3	1995 (14)	1909 (96)	1957 (98)
8 months to < 2 years	3.5	1937 (14)	1838 (95)	1899 (98)
2 years to < 4 years	4.0	1907 (13)	1803 (95)	1866 (98)
4 years to < 6 years	4.5	1864 (13)	1750 (94)	1819 (98)
6 years to < 8 years	5.0	1683 (12)	1542 (92)	1642 (98)
8 years to < 10 years	5.5	1310 (9)	1189 (91)	1279 (98)
10 years to < 12 years	6.0	1248 (9)	1151 (92)	1218 (98)
12 years to < 14 years	6.5	1239 (9)	1110 (90)	1218 (98)
14 years to < 16 years	7.0	1005 (7)	891 (89)	981 (98)

*ID* tube inner diameter; *ETT* endotracheal tube; *SRC* size recommendation chart

**Table 2:** Demographics and clinical characteristics

Parameter	*n* = 14,188
Age, years	5.3 (1.6–9.9)
*Gender*	
Male	8581 (60)
Female	5607 (40)
*Weight, kg*	19.0 (11.2–32.0)
*Height, cm*	112.0 (78.0–142.0)
*ASA physical status*	1.0 (1.0–2.0)
1	7959 (56)
2	4328 (30)
3	1640 (12)
4	259 (2)
5	1 (0)
*Direct laryngoscopic view according to Cormack and Lehane*	
I	12,052 (88)
IIa	1448 (11)
IIb	183 (1)
III	24 (0)
IV	7 (0)
*Final ETT*	
Cuffed	14,179 (100)
Uncuffed*	9 (0)
*Tracheal intubation*	
Nasal	969 (7)
Oral	13,219 (93)
*Day of the week*	
Monday	2607 (18)
Tuesday	2972 (21)
Wednesday	2646 (19)
Thursday	2764 (19)
Friday	2480 (17)
Saturday	403 (3)
Sunday	316 (2)
*Urgency*	
Elective	11,857 (84)
Emergency	2331 (16)

*Uncuffed tubes were excluded per definition of the inclusion and exclusion criteria. The 9 cases of tracheal intubation with uncuffed tubes represent documentation errors that are listed here for transparency

Data presented as median (interquartile range, IQR) or *n* (%)

*ETT* endotracheal tube, *ASA* American Society of Anesthesiologists classification

All data required were extracted from the PDMS which was implemented in 2004 as mandatory documentation tool for all anesthetic procedures in clinical routine. Airway devices used have consistently been documented as preconfigured parameters, using the following distinctive items or menu item: (1) simple (single) intubation vs. reintubation vs. ETT exchange, (2) oral vs. nasal route, (3) cuffed vs. uncuffed ETT, (4) tube sizes and (5) ETT manufacturer. As a core element of any anesthesia record with legal implications, the final ETT chosen has been consistently documented. In cases of multiple or repetitive intubations, only the final tube was documented. Reasons for tube exchange or other comments on airway management were documented as a nonmandatory free text parameter, thus not being directly available for systematic analysis; however, specific assumptions as explained later allow calculation of tube exchange rates for adherence vs. nonadherence to the SRC.

The size of ETTs was considered as correct when the documented final ETT size was identical with the recommended size according to the SRC (SRC-conform as opposed to SRC deviation, Table 1).

Cases documented in the PDMS with the item “reintubation” or “tube exchange” were considered for calculation of the ETT exchange rates. As the reason for ETT exchange was not consistently documented, we used the following assumptions to estimate whether nonfitting was a possible reason for ETT exchange: nonfitting of the primary chosen ETT with SRC-conform size will result in a final tube size with SRC-deviation. The maximum rate is defined by the assumption that all cases with ETT exchange and SRC-deviation result from nonfitting. This is calculated by dividing the number of cases with ETT exchange and SRC-deviation by the number of SRC-conform cases without ETT exchange plus those with ETT exchange and SRC-deviation. As nonfitting according to the SRC is unlikely to be the reason for exchanging an ETT in cases of nasal intubation, the nasal ETTs were excluded. The maximum rate of ETT exchange because of nonfitting in cases with a primary ETT chosen not according to the SRC (SRC-deviation) was calculated as follows: the number of cases with ETT exchange and with an oral SRC-conform final ETT size was divided by the sum of these and the number of cases without ETT exchange and with SRC-deviation of tube size.

### Statistical analysis

Descriptive statistics are presented as either mean ± standard deviation or median (25–75% interquartile range) depending on the distribution of data. Data were modelled by means of a logistic regression, considering tube exchange as the dependent binary variable, and the final ETT size in conformity with the SRC prediction as binary fixed effect. An additional model to further explore the causality between the SRC predicted tube size and the tube exchange rates was constructed. This explored the interaction between the tube size predicted by the SRC and the age (±10%) limit which was affected by the new SRC formula. The null hypothesis, formulated as the absence of an effect for a specific comparison, was assessed by means of a likelihood ratio test considering whether all parameters were equal to zero. Statistical analysis was performed via a fully scripted data management pathway using the R Environment for Statistical Computing Version 4.3.1 (Team RC: A language and environment for statistical computing. Vienna, Austria; 2013). A two-sided *P* < 0.05 was considered to indicate statistical significance.

## Results

From 2009 to 2015, a total of 14,188 children younger than 16 years (median 5.3 years, IQR 1.6–9.9 years) and weighing at least 3 kg (median 19.0 kg, IQR 11.2–32.0 kg) undergoing oral or nasal tracheal intubation using the second generation Microcuff® PET were included. Out of these, 60% were male and 31% of the children were classified as American Society of Anesthesiologists (ASA) physical status 3 or higher. Further details on demographics and clinical characteristics are presented in Table 2.

**Fig. 1:**
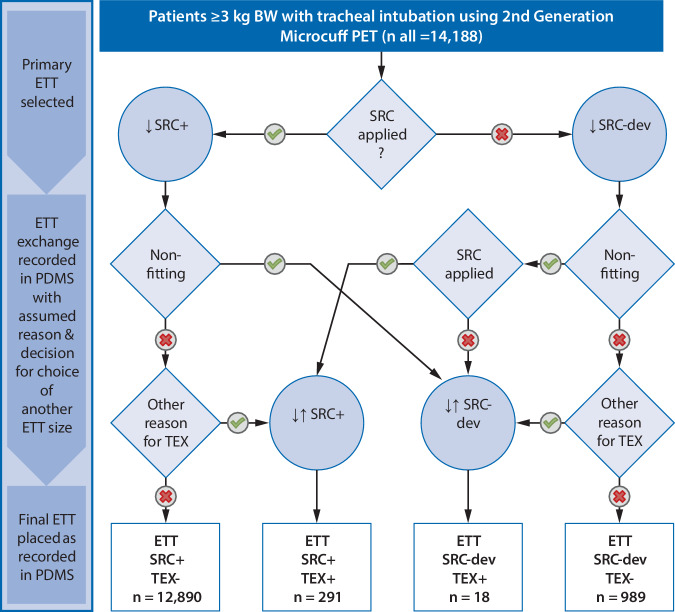
Consort like diagram. Square boxes represent facts documented in the PDMS; decision represented by diamonds and actions represented by circles, based on the assumption that ETT size is only changed during TEX in case of nonfitting of the primary ETT. *OR* operating room**, ***ETT* endotracheal tube, *PDMS* patient data management system, *TEX* documented ETT (tube) exchange, *SRC* size recommendation chart, (for Microcuff pediatric ETT), *SRC-dev* deviation from SRC, *↓* Intubation, *↓↑* ETT exchange

**Fig. 2:**
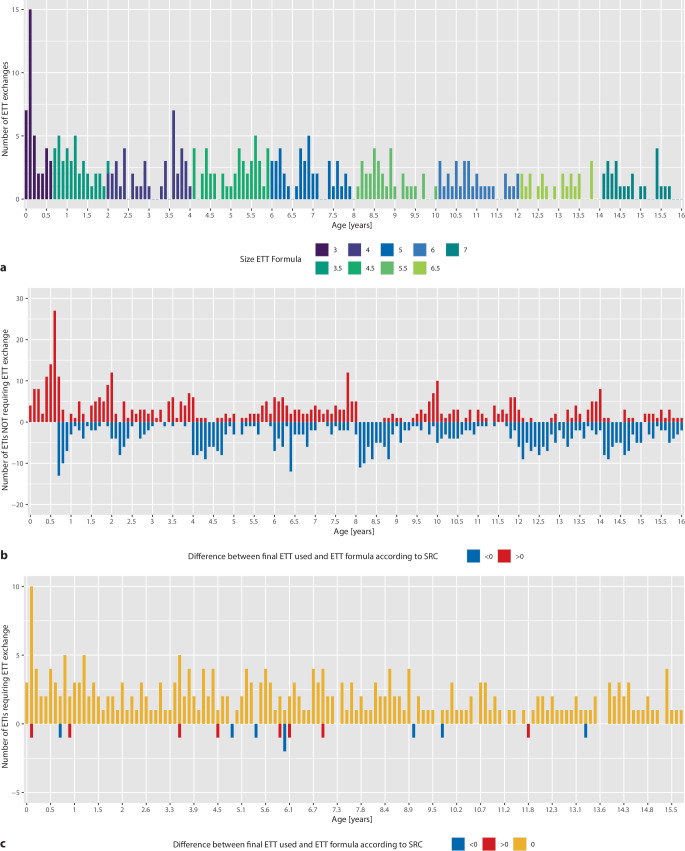
**a** Distribution of ETT exchange rate by age, **b**, **c** discrepancy between ETT selected and the size recommendation according to the manufacturer. **b** Cases not requiring reintubation. **c** Cases requiring reintubation*.* *ETT* endotracheal tube; *SRC* size recommendation chart

The distribution of children intubated, ETT exchange rates and the number of final ETTs according to age groups as defined by manufacturer’s SRC are given in Table 1. The overall number of cases with documented multiple intubations (ETT exchange positive) was 309 (2.2%). Of these, 291 had an appropriate final ETT size according to the SRC and 18 deviated (Fig. 1; Table 3). A separate analysis of the 13,219 oral intubations revealed an ETT exchange rate of 2.1% (*n* = 282, Table 4). Irrespective of ETT exchanges, in 1007 cases (7.1%) the final ETT size documented deviated from the ETT size selection according to the SRC. Figure 2b,c discriminate ETTs smaller or larger for ETT exchange-positive cases or with single intubation related to age.

**Table 3:** Distribution of ETT exchange-positive and ETT exchange-negative intubations (oral and nasal intubations)

	ETT exchange—positive	ETT exchange—negative (single intubation)	Total
*Final ETT size appropriate to SRC*	*291*	*12,890*	*13,181*
*Not appropriate to SRC*	18	989	1007
Smaller than SRC	10	534	544
Larger than SRC	8	455	463
*Total*	*309*	*13,879*	*14,188*

*ETT* endotracheal tube; *SRC* size recommendation chart

**Table 4:** Distribution of ETT exchange-positive and ETT exchange-negative oral intubations

	ETT exchange—positive	ETT exchange—negative (single intubation)	Total
Final ETT size appropriate to SRC	266	12,033	12,299
Not appropriate to SRC	16	904	920
Total	282	12,937	13,219

*ETT* endotracheal tube; *SRC* size recommendation chart

There were multiple peaks for both smaller and larger ETT sizes alongside the borders between neighboring ETT sizes, with a distinct peak for the tube size 3.5mm chosen in children slightly younger than 8 months. There was also a trend towards broader age ranges for children with a smaller ETT size than according to the SRC as compared to children with a larger ETT size than SRC appropriate chosen. Table 3 shows the distribution of final ETT sizes fitting, smaller or larger than according to the SRC, in relation to single or multiple (ETT exchange-positive) intubation. The explicit reason for ETT exchange or multiple intubations is not recorded. Nonfitting as a reason for ETT exchange when the primary chosen ETT was appropriate to the SRC results in a final tube size deviating from SRC and vice versa. This allows the calculation of an assumed maximum rate of nonfitting-induced ETT exchanges when adhering to the SRC as follows:

$$\frac{18}{\left(12890+18\right)}=0.0014\left(0.14{\%}\right)$$

This is the number of ETT exchange-positive cases deviating from the SRC divided by the minimum case number of cases with primary ETT choice appropriate to the SRC. The assumed maximum rate of nonfitting-induced ETT exchange when not adhering to the SRC is as follows (Table 3):

$$\frac{291}{\left(989+291\right)}=0.227\left(22.7{\%}\right)$$

Adjusting for the assumption that non-fitting was not the cause for ETT exchange in those 25 cases with nasal route for the final tube after ETT exchange, the rate is reduced as follows (Table 4):

$$\frac{266}{\left(904+266\right)}=0.227\left(22.7{\%}\right)$$

Further analysis showed no significant influence of nondependent variables and no distinctive differences between ETT exchange-positive or single intubation cases as well as between cases with the final ETT size appropriate to the SRC or not (Table 2). The calculated probability of reintubation for both oral and nasal intubation was 0.14% in adherence to the SRC and 22.73% when not following it. These probabilities amounted to 0.13% and 22.74%, respectively, when only oral cases were included.

Using logistic regression none of the factors depicted in Table 2 had an impact on ETT exchanges or the rate of final ETTs conform with the SRC. The additional model considering the interaction between conformity with the SRC of the final ETT and an age at the changed limit of the SRC, showed an increased odds ratio (OR) for tube exchange of 3.74 (95% confidence interval, CI: 1.13–17.06, *p* = 0.0493). Figure 2b,c shows the discrepancies between tubes that were actually used and the recommendation according to the SRC for cases without (b) and with (c) the need for reintubation.

## Discussion

The aim of this retrospective study was to investigate tube exchange rates using second generation Microcuff® PETs with the manufacturer’s adapted size selection recommendation in a large single institution cohort of children and to identify potential variables associated with an elevated risk of tube exchange.

The main finding of this study is that the probability of reintubation using second generation Microcuff® PETs is significantly lower when the SRC recommendation is followed compared to cases where it is not followed (0.13% vs. 22.73%, *p* < 0.001). The overall ETT exchange rate amounted to 2.2%.

The study results are in line with several previous studies that reported ETT exchange rates between 1.6% and 2.6% for the first generation Microcuff PETs [4, 12, 15]. In all of these studies, between 350 and 1119 children underwent tracheal intubation, which are comparatively smaller cohorts compared to the present study. Nonetheless, all three studies followed the same inclusion criteria for tube exchanges, namely an air leakage above an airway pressure of 20 cmH_2_O with the uninflated cuff.

Assuming low ETT exchange rates as a relevant feature for safe and efficient conductance of pediatric airway management, the presented data underline the clinical feasibility and reliability of the second generation Microcuff® PET and its recommendation for age-related ETT size selection. The data demonstrate that rules for the correct tube size selection according to the SRC are mandatory for the successful application in clinical practice. The small number of children with multiple intubations and a final ETT size not concurrent with the SRC indicates the maximum incidence of a clinical decision to exchange a correctly chosen ETT in the first intubation because the ETT seems too small or too big according to clinical judgement. As this calculated incidence is based on the assumption that nonfitting is the reason for ETT exchange and as there are no data recorded on the real size of the primary tube, this incidence could even be lower. The high number of final fitting ETTs according to the SRC even after multiple intubations if primarily chosen smaller or bigger than recommended and the low ETT exchange rates in adherence to the SRC indicate a potential disadvantage of nonadherence to the SRC. Overall, using the SRC appears to be a clinically more feasible option than alternative assessments, such as ultrasonographic evaluation of the subglottic diameter as previously suggested [18–22].

Multiple reasons can lead to a selection of an ETT size deviating from the SRC, including a random choice of a different size, a deliberate selection of a different tube, and a simple error in choosing the correct tube. The results of this study demonstrate that most cases of deviation from the recommendation are observed at the border between two similar tube sizes and age ranges, which could result from error or deliberate eminence-based choice. The comparatively high deviation rate is surprising as the SRC recommendation for a given ETT size is printed on the packaging of the Microcuff® PET. It is notable that with advancing age, a greater number of ETTs deviate from the SRC (Table 1; Fig. 2b) than in younger age groups. This is unexpected given that the internal diameter of the trachea is typically contingent on the age of the child, rather than on the child’s height or weight [23]. In this context, there is no evident rationale for selecting an ETT other than the one recommended by the SRC, such as for children who are disproportionate in height or weight relative to their age; however, in our institution we frequently encounter children with dysmorphic conditions who are significantly smaller or lighter than expected for their age. It is possible that these children may prompt the anesthesiologist to select a smaller ETT than that recommended by the SRC, particularly as they grow older and the dysmorphic condition becomes more pronounced.

If it is assumed that the majority of cases with multiple intubations and a final ETT size according to the recommendation are due to an initial clinically judged nonfitting tube, the habit of nonadherence to the SRC is deleterious and increased the ETT exchange rate to a maximum of 22.7%. Multiple intubations may increase the risk of airway complications, such as stridor or dyspnea. On the other hand, choosing the right ETT is easy because the recommended age range is printed on the package. A smaller ETT size should however be considered in children with documented prior difficult or small airway/laryngeal/tracheal size or strong clinical indicators [3]. In the present study, there were no significant differences between age groups in terms of the ETT sizes and ETT exchange rates. This shows that the size selection recommendation is valid across the entire age span.

This study has several limitations that must be noted. In principle, the validity of the data is limited due to the retrospective and monocentric nature of the study. Nonetheless, we were able to include a large dataset due to the continuous data acquisition using the PDMS. Underreporting of the ETT exchange rate is possible, which was the primary outcome parameter. The PDMS system used for the analysis only recorded the final ETT size, whereas the numbers or reasons for change were not consistently recorded. Furthermore, no sufficient data were available to capture risk factors for the need of a tube change, e.g., regarding size or body weight especially in case of deviations from the recommendation. Assumptions on probable reasons and constellations have been required to allow a calculation of ETT exchange rates caused by nonfitting PET. The resulting risk ratios are hypothetical but can be used as a maximum rate and thus indicate the worst possible impact of an ETT size selection not according to the SRC. Situations where an uncuffed ETT was required instead of the recommended cETT were not covered using the current study design.

In conclusion, ETT change rates using the second generation Microcuff® PETs with the manufacturer’s adapted recommendation for age-related size selection were consistently low over the whole pediatric age range. This indicates that adequately designed cETTs can be used reliably in combination with the provided SRC in general pediatric anesthesia. Nonadherence to the SRC may significantly increase ETT exchange rates with all its disadvantages and risks. Therefore, we strongly recommend a careful use of well-designed cETTS for daily practice in pediatric anesthesia adherence to the SRC.

## Data Availability

Upon reasonable request, data will be made available.
